# Wet Tap-Induced Spinal Cord Stimulator Trial Failure in Failed Back Surgery Syndrome: A Case Report Highlighting Intrathecal Drug Delivery for Treatment-Resistant Pain

**DOI:** 10.7759/cureus.85793

**Published:** 2025-06-11

**Authors:** Shivang Patel, Matthew Thomas, Harthik Kambhampati, John Stauffer, Tony El-Hayek

**Affiliations:** 1 College of Medicine, Lake Erie College of Osteopathic Medicine, Bradenton, USA; 2 Anesthesiology, Mercy Health - Allen Hospital, Oberlin, USA

**Keywords:** failed back surgery syndrome (fbss), intrathecal pump therapy, neuromodulation therapies, spinal cord stimulation (scs), unintentional dural puncture

## Abstract

A 59-year-old female with failed back surgery syndrome (FBSS) following multiple lumbar surgeries underwent a spinal cord stimulator (SCS) trial for chronic pain management. However, the trial failed due to severe nausea, vomiting, and postural headaches secondary to unintentional dural puncture (wet tap) during lead placement. This resulted in cerebrospinal fluid (CSF) leakage, low-pressure headaches, and subsequent trial intolerance, necessitating early lead removal. Given her failed response to conservative treatments, neuropathic medications, and systemic opioids, an intrathecal pump (ITP) trial was pursued, yielding 90% pain relief and leading to permanent pump implantation. This case highlights the impact of procedural complications on neuromodulation outcomes and the role of intrathecal drug delivery as an alternative in patients who cannot tolerate SCS.

## Introduction

Chronic back pain is a common condition that can significantly limit mobility, interfere with activities of daily living (ADLs), and reduce overall quality of life. Studies estimate that it affects between 51% and 84% of adults [1]. Initial management is typically conservative, involving physical therapy, pharmacologic treatments, and lifestyle modifications. Interventional injections or surgery are often reserved for refractory or complex cases.

However, some patients continue to experience persistent pain even after multiple surgeries. This condition is known as failed back surgery syndrome (FBSS), first characterized by North et al. in 1991 [2]. The International Association for the Study of Pain defines FBSS as “lumbar spinal pain of unknown origin either persisting despite surgical intervention or appearing after surgical intervention for spinal pain originally in the same topographical location” [3]. Its incidence is difficult to determine due to its varied causes, but estimates suggest it affects 10% to 40% of post-surgical patients [4].

The etiology of FBSS is multifactorial, with contributing factors across the preoperative, intraoperative, and postoperative periods. These include psychological comorbidities, poor patient selection, suboptimal surgical technique, recurrent herniation, and adjacent segment degeneration [4].

FBSS can be managed with medications such as nonsteroidal anti-inflammatory drugs (NSAIDs), acetaminophen, antidepressants, antiepileptics, muscle relaxants, and opioids [5]. A multidisciplinary approach, including physical rehabilitation and psychological support, has also shown benefit [5]. Epidural steroid injections (ESIs) are sometimes used for temporary relief by reducing radicular inflammation, but their effects are often short-lived [6,7]. For patients with persistent pain despite conservative measures, spinal cord stimulation (SCS) and intrathecal drug delivery via intrathecal pumps (ITPs) are advanced treatment options [8].

We present a case of a 59-year-old female with FBSS whose SCS trial failed due to an unintentional dural puncture. Her case demonstrates the impact of procedural complications on neuromodulation outcomes and highlights intrathecal drug delivery as a viable alternative for treatment-resistant pain.

## Case presentation

A 59-year-old female with a history of chronic low back pain following five lumbar spine surgeries (beginning in the 1980s) presented with persistent, debilitating pain consistent with FBSS. Her pain limited mobility and ADLs, with a reported score of ≥6/10 during activity.

She had previously been evaluated by multiple pain specialists and had undergone extensive conservative treatments with minimal or no relief. Neuropathic agents such as pregabalin and topiramate offered little benefit. Opioid therapy, including methadone and other agents, was associated with inadequate response, side effects such as sedation, nausea, and dizziness, as well as poor oral tolerance characterized by gastrointestinal upset and poor appetite. NSAIDs and muscle relaxants were ineffective. Multiple ESIs were also attempted, but they resulted in elevated blood glucose levels and no lasting pain relief.

An SCS trial was initially planned; however, the procedure failed due to an unintentional dural puncture (wet tap), resulting in severe postural headaches, nausea, and vomiting. The patient was unable to tolerate the trial, and it was ultimately deemed unsuccessful. Due to ongoing refractory pain, an ITP trial was recommended.

On initial evaluation at our clinic, the patient described severe lumbar pain, occasionally radiating to the lower extremities but predominantly localized to the low back. Physical examination revealed no worsening of pain with flexion, extension, or rotation. Thoracic MRI demonstrated mild canal stenosis at T8-T9, moderate stenosis at T10-T11 secondary to a 3 mm left paracentral disc protrusion, and additional mild multilevel degenerative changes. Lumbar MRI showed prior posterior lumbar interbody fusion at L5-S1, mild-to-moderate right neural foraminal narrowing at L5-S1 due to osteophyte formation, and no other significant findings.

A caudal ESI performed on 9/26/24 resulted in 0% pain relief, confirming a negative response. On 11/21/24, an intrathecal morphine trial (0.3 mg) was performed and provided 90% pain relief over 24 hours. Based on this positive response, the patient underwent permanent ITP placement (Medtronic SynchroMed III (Medtronic, Inc., Minneapolis, MN, USA), right abdominal site) on 1/29/25.

An IV was placed, and the patient was positioned in the left lateral decubitus position with proper padding and spinal alignment. After sterile preparation, the L3-L4 interlaminar space was identified under fluoroscopy. The skin was anesthetized with 2% preservative-free lidocaine, and a small incision was made. A 14-gauge Touhy needle was advanced into the intrathecal space, with positive CSF flow confirmed on fluoroscopy. An intrathecal catheter was inserted and advanced to the T9 vertebral level.

A subcutaneous pocket was created in the right lower abdomen, and the catheter was tunneled and connected to the ITP. The pump was anchored securely and filled with preservative-free normal saline due to temporary pharmacy limitations. All wounds were irrigated and closed with absorbable and nylon sutures. The patient tolerated the procedure well, maintained stable motor strength, and was discharged home in stable condition. Follow-up is scheduled in one to two weeks for a wound check and administration of intrathecal pain medication.

**Figure 1:**
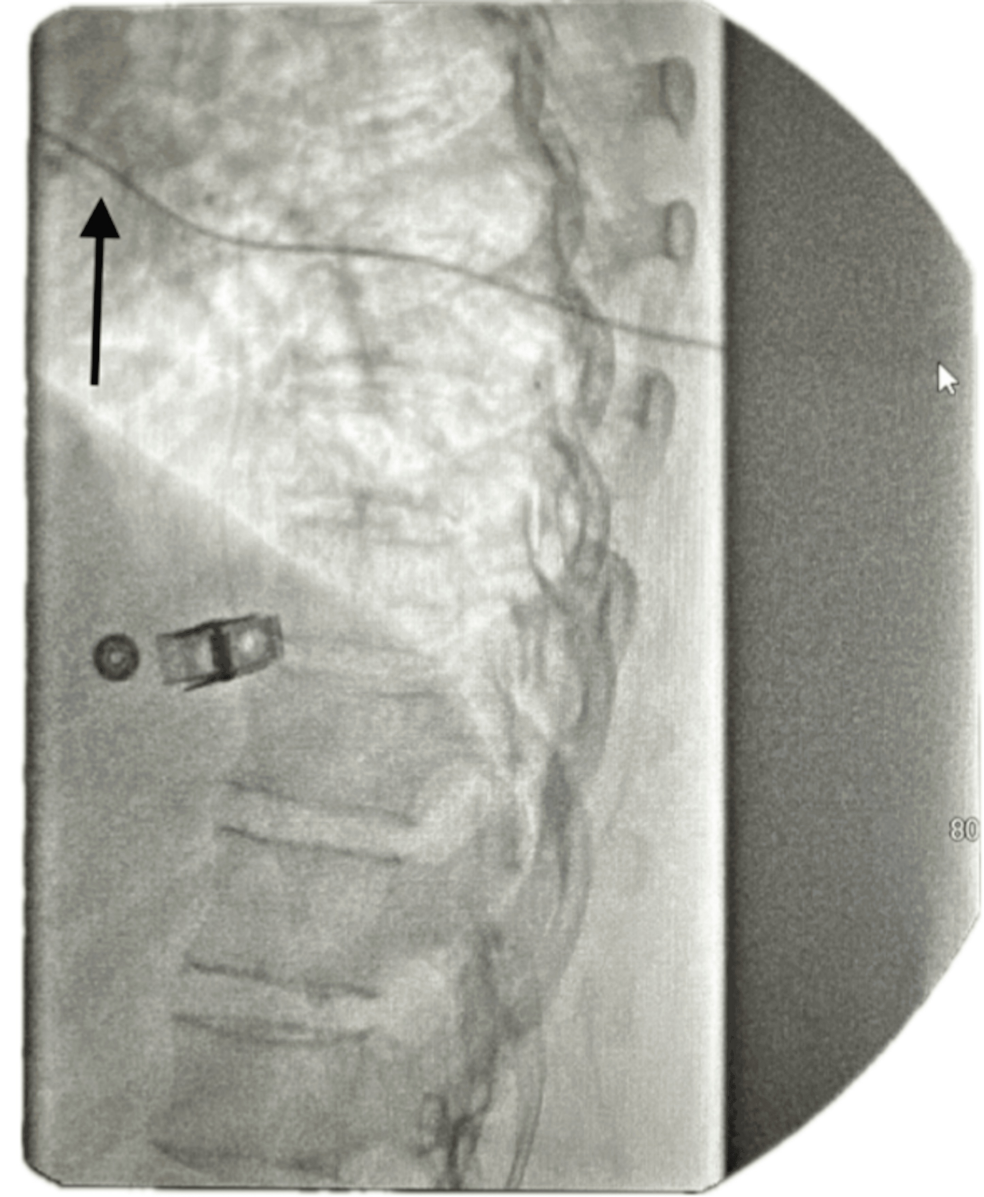
Lateral lumbar X-ray view demonstrating intrathecal catheter tip placement. The black arrow on the left highlights the path of the catheter extending toward the spinal canal.

The ITP is programmed to deliver a micro-dose regimen consisting of morphine 2 mg and bupivacaine 2 mg (0.5%) in a total volume of 20 mL. The infusion rate is set at 0.7496 mg/day. The catheter tip is positioned at the T8 level within the intrathecal space, as confirmed by imaging (Figure 1). The low reservoir alarm is set to trigger at 2.0 mL to ensure timely refilling and management.

Post-implantation, she reported significant pain reduction, increased ability to perform ADLs, and decreased reliance on systemic opioids and neuropathic medications.

At subsequent follow-ups, she continued to experience durable pain relief, improved mobility, and enhanced quality of life. No complications or device-related side effects were reported. Given her history of treatment-resistant pain and failure of systemic therapies, intrathecal drug delivery was a successful alternative following a failed SCS trial.

## Discussion

SCS is a neuromodulation therapy used to manage chronic neuropathic pain by delivering electrical impulses to the spinal cord’s dorsal column, which processes sensory inputs such as touch, vibration, and proprioception [9]. This therapy is based on the gate control theory of pain, which states that stimulating non-painful nerves can inhibit pain signals [9].

The trial phase of SCS is pivotal before recommending permanent implantation, as it confirms the therapy’s ability to provide meaningful pain relief before making a long-term commitment. During the trial, temporary percutaneous leads are placed epidurally, and patients assess their pain relief over several days. If pain reduction exceeds 50%, permanent implantation is typically pursued [10]. This process helps identify responders, minimizes unnecessary surgery, and ensures realistic expectations. Trial success is a strong predictor of long-term outcomes.

While SCS has many benefits, complications are not uncommon and must be considered in patient selection. Overall complication rates for SCS range from 31.9% to 43%, though most are not severe [11]. The most frequently reported complications include lead migration, infection, hardware malfunction, CSF leakage, and wet tap. Although wet taps are rare, one retrospective study found an incidence of postdural puncture headache in 0.81% of lead placements (6 out of 745 cases) [12].

A wet tap is an inadvertent dural puncture during percutaneous lead placement. This leads to CSF leakage, resulting in intracranial hypotension and symptoms such as postural headache, nausea, and dizziness [13]. These symptoms are often pathognomonic and may compromise a patient’s ability to complete the trial. As a result, false-negative outcomes may occur, leading to inappropriate discontinuation of SCS therapy [14].

In this case, the SCS trial failed due to intolerance caused by wet tap-related symptoms. It is essential to differentiate between procedural complications and actual therapy failure. Misattributing trial failure to the ineffectiveness of SCS may deny patients access to a potentially beneficial therapy. The wet tap itself is not a failure of neuromodulation; rather, it is a correctable procedural issue. In some cases, placing a blood patch and repeating the trial may salvage the opportunity for therapy success [13].

Modern advancements such as closed-loop SCS systems allow for real-time adjustments based on neural feedback and may improve outcomes despite initial complications [15]. However, in this case, a change in treatment strategy was made without the opportunity to apply an epidural blood patch or repeat the trial. This highlights the need for careful evaluation before declaring a trial unsuccessful.

Given the patient’s refractory pain and intolerance to the SCS trial, intrathecal drug delivery was pursued. This technique involves the direct administration of medication into the CSF through an implanted pump and catheter, bypassing the blood-brain barrier [16]. This method improves drug bioavailability and enables effective pain control with smaller doses compared to systemic administration.

A survey of 533 patients with ITPs found that 431 reported significant pain relief, 95 experienced some relief, and only seven had no benefit [17]. A wide range of medications may be delivered intrathecally, including opioids, local anesthetics, baclofen, and ziconotide. In this case, morphine was used, though other opioids such as hydromorphone or fentanyl are also common.

ITPs deliver medication at a continuous basal rate and may provide bolus dosing for breakthrough pain [17]. Pump placement is typically in the abdomen but may vary based on patient factors. In this case, the pump was implanted in the right abdomen.

Refills are required approximately every one to three months, depending on the drug concentration and dose. Refills are performed in a clinical setting via direct access to the pump reservoir [16]. Regular follow-up and dose adjustments are essential to optimize pain relief and minimize complications [17].

Most patients initiating ITP therapy are already on systemic opioids. Tapering systemic opioids by approximately 50% before ITP placement is recommended to avoid withdrawal symptoms and reduce the risk of opioid toxicity from dual routes of administration [17]. In this case, the patient’s previous opioid regimen was tapered prior to implantation. The transition was closely monitored to ensure safety and efficacy.

This case highlights the importance of distinguishing between procedural complications and therapy failure when evaluating patients with FBSS. While less invasive options should be explored first, both SCS and ITP remain valuable tools in the treatment of refractory pain. Recognizing and appropriately managing procedural complications, such as a wet tap, can prevent the premature abandonment of effective interventions.

## Conclusions

This case highlights the importance of thoroughly evaluating treatment options in patients with FBSS, particularly when complications such as a wet tap threaten the success of an SCS trial. It underscores the need to distinguish between true treatment failure and procedure-related complications, as premature abandonment of SCS therapy may prevent patients from receiving effective pain relief.

Additionally, this case demonstrates that intrathecal drug delivery through an ITP can provide significant pain reduction and functional improvement in patients who are nonresponsive or unable to tolerate other forms of neuromodulation. By delivering medication directly to the spinal cord, this method offers a viable and effective option for managing chronic, treatment-resistant pain in patients with FBSS.
